# Supplementary material for: Are carbon emissions trading and green financial instruments synergistic? -Comprehensive quantitative research based on content analysis
Source: PLoS One. 2024 Mar 7;19(3):e0298601. doi: 10.1371/journal.pone.0298601 (PMC10919601; doi:10.1371/journal.pone.0298601)
Supplement: S1 Data — (ZIP) [file pone.0298601.s001.zip › Data/地级市Z_F工作报告.zip!/╕ú╜¿/╕ú╓▌╩╨/└┤╫╘╣1⁄2╓┌║┼í╛┬φ┐╦╩2╛▌═°í┐_2010.docx]

各位代表：

　　现在，我代表福州市人民政府向大会作政府工作报告，请予审议，并请市政协各位委员和其他列席人员提出意见。

一、2009年工作回顾

　　2009年是很不平凡的一年。在省委、省政府和市委的正确领导下，我市各级政府认真贯彻落实党的十七大和十七届三中、四中全会精神，以深入学习实践科学发展观和庆祝新中国成立60周年为动力，紧紧抓住中央支持福建加快海西建设的机遇，积极有效应对国际金融危机，全力以赴保增长、保民生、保稳定，较好地完成了市十三届人大四次会议确定的目标任务。据初步统计，全市生产总值达2520.69亿元，比增12.6%；地方级财政收入达195.26亿元，比增15.6%；全社会固定资产投资完成1623.6亿元，比增30%；出口总额达115.82亿美元，比降14.8%；实际利用外资按验资口径达10.3亿美元，超过预期目标2.3亿美元；社会消费品零售总额达1332亿元，比增17.7%；居民消费价格总水平比降0.9%；城镇居民人均可支配收入达20316元，实际增长9.9%；农民人均纯收入达7702元，实际增长9.1%；城镇登记失业率为3.3%；人口自然增长率为5.7‰。单位地区生产总值能耗下降2.75%、二氧化硫排放量减少12%、化学需氧量减少0.1%的节能减排目标预计可以完成。一年来的主要工作和成效是：

　　（一）实践海西，发展思路有提升。国家、省出台加快海西建设的《若干意见》、《实施意见》后，我市主动对接，明确提出融入整体、服务大局、发挥优势、加快发展的一系列思路，从更高的站位、更宽的视野谋划福州发展。一是对福州发展定位的认识有了提升。充分认识到福州作为省会，在海西发展中具有举足轻重的作用，更加注重把福州的发展摆进福建的全局、海西的全局中去定位、去谋划、去突破，进一步激发和调动全市上下推进发展的积极性、创造性，形成谋发展、促发展、抓发展的氛围。二是对福州发展优势的认识有了提升。充分认识到福州作为省会独具全省政治、经济、文化中心地位，央属、省属资源丰富，侨台、港口、人才、生态等优势明显，更加注重用好、用活、用足优势，开拓发展空间，提升发展层次和品位，进一步走出具有福州特色的发展新路。三是对福州发展基础的认识有了提升。充分认识到加快福州发展的当务之急是加快产业发展，更加注重在已有的发展基础上，做大做强做优三次产业，下力气引进产业龙头项目，扶持高成长性企业发展，培育新经济增长点，提高产品市场占有率，进一步夯实发展基础、增强发展后劲。四是对福州发展载体的认识有了提升。充分认识到发挥福州在海西建设全局中的带动和辐射作用，必须高起点、高标准、高层次、高水平推进城市建设，更加注重树立精品理念、特色理念，做好城市规划，拓展城市发展空间，进一步提升城市发展水平。五是对福州发展环境的认识有了提升。充分认识到抢占区域竞争制高点重在营造良好的发展环境，更加注重精心打造投资、休闲、宜居环境，提高政府的执行力、公信力、亲和力，进一步促进创造财富的各类要素在福州集聚涌流。

　　（二）应对危机，持续增长有成效。为积极应对国际金融危机的挑战和冲击，我市认真贯彻落实中央、省关于扩大内需、促进经济平稳较快发展的一揽子政策，迅速制定出台支持工业产品开拓市场、扶持高成长性企业发展、鼓励重点外贸企业扩大出口、帮助企业减轻负担等措施，据统计，市本级财政为扩内需保增长投入资金64亿元，出台各项税费减免和补助政策减轻企业负担3.2亿元。一是农业和农村经济稳步发展。全市农林牧渔业总产值达409.84亿元，比增5%；粮食生产保持稳定，水产、果蔬、食用菌、茶叶、竹木等特色优势产业发展加快；209家农业产业化龙头企业年产值达337.1亿元，比增12.2%，带动农户85.3万户。二是工业生产逐月回升。规模以上工业增加值达906.6亿元，比增14.1%；197项工业新增长点项目新增产值195亿元，南北“两翼”对工业增长贡献率达43.2%，戴姆勒汽车、德盛镍合金等50项重大工业项目建成投产。三是高新技术产业增势强劲。实现高新技术产业工业产值1503亿元，比增20%，瑞芯、网龙、福大自动化等一批企业已走在全国同行业前列，福州申报国家动画产业基地通过验收。四是自主创新能力进一步提高。新设立8家省级企业技术中心，新成立6家行业技术创新中心，新大陆科技集团被评为国家级创新型企业，福州在全省率先成为国家知识产权工作示范城市，并连续第8次获得全国科技进步先进市称号。五是现代服务业提速发展。海峡水产品、农副产品、汽车等交易中心动工建设，成功举办第二届亚太批发市场大会暨第三届中国（福州）国际农产品贸易对接会等6场国家级会展活动。六是消费对经济增长的拉动作用进一步增强。住宅、旅游等消费持续升温，落实家电、汽车下乡及以旧换新等政策成效明显，“万村千乡”市场工程建设扎实推进，农村市场消费增速首次快于城市。

　　（三）项目带动，发展后劲有增强。立足扩大内需，强化项目带动，实施市级重点项目289项，完成投资561.7亿元。中央4批228项新增投资项目完成年度投资任务。向社会公开承诺的在国庆、元旦前竣工、开工的57项重大项目建设任务如期完成。温福、福厦高速铁路建成通车，福州发展跨入高铁时代。动工建设福永高速公路、福泉高速公路福州段扩建等一批项目，全市在建高速公路里程达254.48公里。福州港新建成5万吨级以上泊位4个，港口货物吞吐量达8084.3万吨、集装箱吞吐量达122.2万标箱。福州（长乐）国际机场年旅客吞吐量突破500万人次。福清核电站建设加快推进，三山嘉儒风电场、高山风电场建成投产。轨道交通1号线动工建设。（四）精心打造，城市面貌有改变。开展了新一轮城市总体规划修编和会展岛等重点区域城市设计。海峡国际会展中心、火车南站建设及火车北站改扩建等拓展新区的重大项目进展顺利。海峡金融商务区动工建设。首批500万平方米旧城区及危旧房（棚屋区）改造开始启动。完成茶亭街主干道、福峡路、福湾路、杨桥路等24项路桥工程建设，改造提升西二环等3条主干道。新辟国货路等3条公交专用道，新增更新环保公交车704辆，公交出行率达22.1%。城区环卫机械化清扫率从28%提高到48%。实施西湖、左海截污工程，西湖和内河水质明显改善。大学新区防洪排涝主体工程基本完成。城区主干电网及电缆下地工程启动实施。完成鼓楼、台江、晋安等城区天然气气源转换。集中清理中心城区违法建设和综合整治鼓岭取得初步成效，有力遏制了违法建设的势头。拆除违规违章设置的户外广告牌2500面，完成城区836幢建筑物立面整治，治理乱张贴等城市管理专项整治行动成效明显。建成开放西湖公园大梦松声景区、环西湖左海步行道、乌山北坡公园、鼓楼前公园，改造提升茶亭公园、屏山公园、左海公园、温泉公园，新增城市公共绿地面积60万平方米。

　　（五）积极探索，开放改革有作为。设立福州（平潭）综合实验区，规划编制、招商引资、资金筹措等工作扎实推进，平潭环岛路等一批重大基础设施建设开始启动。成功举办第十一届“5·18”海交会和首届海峡论坛·县市主题日、海峡渔业周暨第四届渔博会等重大活动。实现福州至台湾本岛空中双向直航常态化和海上客运直航。福州商业银行更名为福建海峡银行。新大陆科技集团成为大陆首家经正式核准的入台投资企业。海峡两岸合唱节等特色活动形成品牌效应。招商引资质量进一步提高，新引进33项千万美元以上大项目和4家世界500强企业，合同外资达8.19亿美元，高新技术产业、现代服务业利用外资占全市合同外资总额的54%。体制机制创新力度加大，公共资源市场化配置项目向基础设施、公共服务等领域拓展。以四个投融资平台、六个产业集团、六个专业公司为主构架的国资运营格局初步形成。交建公司、建工集团顺利发行企业债券填补了我市国有企业债券融资的空白。华映光电、上润精密、网讯科技、连江远洋等企业在境内外成功上市。民营经济持续健康发展。政府机构改革稳步推进。农村综合改革不断深化。财税、科技、教育、文化、卫生、价格等领域的各项改革迈出新步伐。榕港榕澳合作、闽东北五市区域协作、山海协作、海内外友城合作以及侨务、外事、异地商会、对口援建等工作取得新成效。

　　（六）统筹兼顾，社会事业有发展。义务教育免除学杂费等政策全面落实，12个县（市）区实现“双高普九”，高中阶段毛入学率达98.2%，鼓楼区被评为全国推进义务教育均衡发展工作先进地区。新建6所中小学，全面完成中小学校舍安全排查鉴定。职业教育加快发展，民办教育、学前教育规范化管理切实加强，农民工子女入学难等问题得到较好解决。医疗卫生服务水平进一步提升，市二医院急救中心大楼、传染病院新病房大楼、儿童医院门诊裙楼投入使用。甲型H1N1流感等重大疾病得到有效控制。社会福利、救助和慈善事业加快发展，市红十字会获得全国红十字会先进单位称号。建成市艺校新校区一期工程，成功举办庆祝新中国成立60周年文艺晚会等大型文化活动，“读书月”、“激情广场”等群众性文化活动蓬勃开展，电视剧《郑和下西洋》等一批文艺创作精品获得国家级大奖，在第四届省艺术节上我市获奖总数居全省首位。完成林则徐纪念馆改扩建和二梅书屋、林聪彝故居修复，“三坊七巷”入选中国十大历史文化名街。积极培育文化旅游品牌，鼓山、于山、“三坊七巷”创建国家4A级旅游景区通过验收。全民健身活动广泛开展，在第十一届全运会上我市运动员取得了7金3银5铜的优异成绩。创建文明城市工作取得新成果，福州获得全国未成年人思想道德建设工作先进城市称号，城市公共文明指数测评成绩居30个省会、副省级城市第七位。人口计生工作取得新成绩，低生育水平保持稳定，出生人口素质进一步提高。民族宗教、新闻出版、广播电视、社会科学、地方志、档案、老龄、妇女儿童、残疾人等各项事业全面发展。

　　（七）攻坚克难，生态环境有改善。着力推进节能减排、环境综合治理工作，积极组织实施了陶瓷企业改燃气降耗减排、电力企业烟气脱硫改造、绿色照明等重点工程。建成闽侯城关等3家污水处理厂，动工建设连坂污水处理厂等6家城镇污水处理厂和福清垃圾焚烧厂，城市污水处理率达81%，生活垃圾无害化处理率达98.1%。城区空气质量优良率达96.7%，区域环境噪声、交通噪声均优于国家规定标准。闽江南北港、敖江、龙江等重点流域水环境综合整治成效明显，饮用水源水质优于国家标准。闽江下游水域清理整治取得阶段性成效，非法采砂、“连家船”得到有效治理。福州入选全国可再生能源建筑应用示范城市，创建全国绿化模范城市工作通过国家核查，生态环境质量及城市环境综合整治定量考核成绩持续位居全省前列。　（八）着力民生，惠民实事有落实。年初确定的16件57项为民办实事项目基本得到落实，取消公交车空调收费、提高村主干补助标准等一批惠民措施深受欢迎。农村生产生活条件进一步改善，完成17座病险水库除险加固、11.06公里海堤强化加固，解决农村25.63万人饮水安全问题，实施“造福工程”搬迁5000人，新建改建农村公路402.8公里、农村寄宿制学校生活用房4.19万平方米、户用沼气池3820口、农家书屋486家、农村敬老院6所、农民体育健身工程点345个、农民体育健身活动中心14个，改造提升乡镇综合文化站30个、乡镇卫生院25所。城乡就业保持稳定，城镇新增就业15.23万人，实现农业富余劳动力转移就业5.51万人。社会保险覆盖面不断扩大，企业退休人员基本养老金和城镇职工、居民基本医疗保险待遇持续提高，在榕大学生全部纳入城镇居民基本医疗保险范围，城镇职工和居民基本医疗保险累计参保率达96.2%，新型农村合作医疗参合率达95.3%。低收入家庭生活保障、廉租住房保障得到落实。居家养老服务在城区全面推行，医疗救助全面覆盖城乡困难群众。侨房腾退任务基本完成。“餐桌污染”得到有效治理，主要食品安全检测指标全部达标。“平安福州”建设不断深化，人民群众对社会治安满意率达94.5%。安全生产、信访、行政复议、矛盾纠纷排查调处、人民调解、法律援助、法律服务以及国防动员、双拥共建、民兵预备役、海防、人防等工作进一步提升，省会城市保持和谐稳定。

　　（九）转变作风，政府效能有提高。坚持依法行政，提请市人大审议地方性法规草案2件，制定政府规章和规范性文件25件。切实规范行政处罚自由裁量权，细化全市行政处罚权5399项。修订完善了市政府工作规则。机关效能建设活动深入开展，绩效管理、行政问责全面推行，行政审批进一步提速。电子政务建设水平持续提升，“中国福州”门户网站在全国省会及计划单列市政府网站中的排名跃居第六位，便民呼叫中心12345系统获得中国十佳电子政务公共服务优秀应用案例称号。廉政建设扎实推进，行政监察、审计监督、纠正部门和行业不正之风、清理“小金库”以及政务公开、政府信息公开等工作取得新成效，集网上行政审批、行政处罚、公共资源交易、中介诚信管理、行政监察于一体的行政权力阳光运行平台建成投入使用，市审计局首次被评为全国审计系统先进集体。认真执行人大及其常委会决议决定，自觉接受人大法律监督、工作监督和政协民主监督，广泛听取各民主党派、工商联、人民团体和社会各界人士意见建议，办复576件省、市人大代表建议、批评、意见和583件省、市政协委员提案，满意率分别达95.7%和99.5%。

　　各位代表，回顾过去一年的工作，我们取得的成绩来之不易。这是在省委、省政府和市委正确领导下，全市人民团结拼搏、攻坚克难，社会各界人士大力支持、热情帮助的结果。在此，我代表市政府，向全市人民，向在榕工作的外来建设者、外籍人士，向给予政府工作有力支持与有效监督的人大代表、政协委员和各民主党派、工商联、各人民团体、无党派人士、离退休老同志和社会各界人士，向为福州改革发展稳定做出积极贡献的省直机关企事业单位和驻榕部队、武警官兵、公安政法干警，向关心帮助福州建设发展的香港特别行政区同胞、澳门特别行政区同胞、台湾同胞、海外侨胞、国际友人，表示衷心的感谢！

　　在看到成绩的同时，我们也清醒地认识到，福州综合实力、核心竞争力和发展后劲离经济社会发展新形势的要求还有较大差距。一是福州产业基础仍较薄弱，市场占有率和竞争力不强等问题仍较突出，经济回升的基础还不稳固，外贸出口未能实现预期目标；二是来榕投资大的产业项目不多，尤其是能形成新增长点的大项目不多，项目落地的配套设施建设明显滞后；三是城乡发展、区域发展不平衡，部分县乡财政较困难，农村规划建设滞后以及农民持续增收问题还没有得到很好解决；四是城市建设精品不多，市区交通拥堵等状况仍较突出，违法建设时有发生；五是省会人才、科教优势未得到充分利用和发挥，自主创新能力不够强，拥有自主知识产权的产品比例还不高；六是环境保护、生态建设任务较重，资源节约集约利用水平不高；七是教育、医疗、住房、就业、社会保障等社会事业和民生领域要解决的问题还比较多；八是政府工作效能、服务水平、服务态度离人民群众的要求还有较大差距，个别部门和工作人员效能低下、敷衍塞责、推诿扯皮、吃拿卡要等问题还时有发生。对这些问题，我们应认真加以解决。二、2010年工作安排

　　各位代表，2010年是实施“十一五”规划的最后一年,也是在新的起点上全面推进海西建设的重要一年。总体上看，今年经济发展环境将好于去年，但经济发展中的不确定、不可预料的因素增多，保持平稳较快发展的任务还很艰巨。我们要把握发展机遇，融入发展大局，在发展中促转变，在转变中谋发展，更加有效应对国际金融危机的冲击，进一步巩固经济回升的基础，全面完成“十一五”目标任务，为“十二五”规划启动实施作出新贡献。

　　根据市委统一部署，今年政府工作的总体要求是：全面贯彻党的十七大和十七届三中、四中全会精神，以邓小平理论和“三个代表”重要思想为指导，深入贯彻落实科学发展观，按照“五个更加注重”的要求和建设海峡西岸经济区的战略部署，紧紧抓住发展第一要务，进一步加速度、强投入、调结构、鼓干劲、上水平，在扩大需求中着力推动持续增长，在转变方式中着力提升发展质量，在先行先试中着力深化改革开放，在改善民生中着力促进社会和谐，打造宜居城市，努力在建设海峡西岸经济区中发挥省会中心城市的带动和辐射作用。

　　初步安排2010年全市经济社会发展的主要预期目标是：地区生产总值增长12%；地方级财政收入增长12%；全社会固定资产投资增长30%；出口总额增长10%；实际利用外资按验资口径达103亿美元；社会消费品零售总额增长16%；居民消费价格总水平涨幅控制在3%以内；城镇居民人均可支配收入实际增长8%，农民人均纯收入实际增长6%；城镇登记失业率控制在4%以内；人口自然增长率控制在7‰以内；单位地区生产总值能耗下降2.75%，二氧化硫排放量减少0.1%，化学需氧量减少0.1%。

　　为实现上述目标，今年政府工作必须把保持经济平稳较快发展与结构调整结合起来，使经济增长建立在结构优化的基础上；必须把扩大内需特别是消费需求与稳定外需结合起来，促进经济在更高水平上均衡发展；必须把统筹城乡区域协调发展与推进城镇化结合起来，加快形成新的增长极；必须把推动自主创新与培育战略性新兴产业结合起来，走出创新驱动的发展新路；必须把深化改革与促进发展结合起来，从根本上推动发展方式转变和经济结构调整；必须把发展经济与改善民生结合起来，让最广大人民群众更多地共享发展成果。重点抓好以下十个方面的工作：

　　（一）把“三农”工作作为发展基础抓紧抓实

　　一是重视对农业的投入，促进农业增效、农民增收。完善农业支持保护制度，增加涉农补贴规模，推进政策性农业保险试点。落实鼓励农民种粮各项政策，稳定粮食生产，防止耕地抛荒，抓好粮食储备和流通，保障粮食安全。加强“菜篮子”工程建设，稳定农产品价格，拓展农民增收渠道，丰富人民群众餐桌食品。

　　二是加快发展现代农业，提升农业产业化规模。以高产、优质、高效、生态、安全为导向，壮大水产、果蔬、茶叶、食用菌、竹木等特色优势产业，加快发展设施农业、休闲观光农业等新兴农业。大力推进林业十大工程建设，提升林业生产质量和效益。发挥海洋优势，扶持发展远洋渔业，拓展海湾外养殖，大力发展以水产品加工为重点的农产品精深加工，推进海西（连江）水产品加工等三大基地建设。实施“强龙带动”战略，做大做强农业产业化龙头企业。发展品牌农业，推行标准化生产，严格农产品质量安全监管，加强农产品注册商标、地理标志产品的申报、保护与宣传。加大农业“五新”引进和推广力度，完善农业技术服务体系。

　　三是强化农业农村基础设施建设，持续改善农村生产生活条件。加强农田水利基础设施建设，推进土地整理复垦开发，实施标准化养殖池塘改造8000亩、中低产田改造7600亩，抓好15座中小型水库、11.95公里海堤除险加固工作，治理水土流失面积10平方公里。严密防控动物疫病、农业有害生物和森林病虫害，提高预防和抵御自然灾害的能力。滚动实施新农村建设“双百工程”、农村“家园清洁行动”，建设农村公路280公里，解决农村20万人饮水安全问题，建设户用沼气池3000口，改厕1.1万户，抓好100个以上村的村庄规划。

　　四是加强农村公共服务能力建设，完善农业社会化服务体系。稳定和完善农村基本经营制度，稳妥推进土地承包经营权流转，发展各种形式的适度规模经营。深化集体林权等农村产权制度改革，完善农村工作新机制，增强农村发展活力。推进农村信用社改制试点，发展村镇银行等新型农村金融机构和农村保险事业。加强“六大员”等农村基层队伍建设，扶持发展农民专业合作组织和乡镇“三农”服务中心、村级综合服务站。认真抓好扶贫开发、对口帮扶、库区移民后期扶持等工作，深入开展“海西春风·春雨·光彩”活动，实施“造福工程”搬迁3000人，对新一轮农村有劳动能力的低保对象1.02万人实行全面帮扶。

　　（二）把第二产业作为发展支撑做强做大

　　一是扶大扶强，支持重点产业和高成长性企业发展壮大。推进机械制造、纺织服装、电子信息、轻工食品、冶金建材、石油化工、生物医药、新材料及能源等八大重点产业调整与振兴发展，抓好华东船厂30万吨修造船、青口镁合金、恒逸化工等58项产业调整与振兴发展重点项目建设。实施德盛特钢等180项工业新增长点项目，争取新增产值500亿元。集中投入加大对高成长性企业扶持力度，着力培育若干家产值超百亿的龙头企业。积极引导建筑企业整合资源，创造条件晋升资质等级，加快培育一批建筑业大型企业和骨干施工队伍。

　　二是以港兴市，支持临港工业加快发展。加快福州港建设发展步伐，推进在建的7个5万吨级以上泊位以及港口集疏运通道、航道工程、口岸查验设施建设，新开工建设7个深水泊位。争取罗源湾港区正式对外开放，大力拓展福州港班轮航线和长乐空港航线，增强港口集聚辐射能力。创新市、县两级投资机制，集中力量加快完善“两翼”地区道路、水电气、污水处理等基础设施，促进石化、能源、冶金、船舶、装备制造等临港工业大项目尽快落地。积极构建江阴、环罗源湾、闽江口南翼等产业集聚区，加快台商投资区和国家级开发区扩区，在罗源湾南北两岸和福清江阴等地各开发工业用地5000亩以上，在马尾（琅岐）、长乐空港、闽侯等地各开发工业用地3000亩以上，培育铝深加工产业园、不锈钢产业园、石化专区等一批特色产业园区。

　　三是精心谋划，支持高新企业、新兴产业快速发展。认真抓好海西高新技术产业园、福州软件园建设与招商，争取引进落地一批高技术含量、高成长性的新项目。做大做强光电及核电、风电等新能源产业，加快发展节能环保产业，构建海西新能源、环保产业基地。大力发展海洋经济，推进八大“蓝色产业”发展壮大。培育生物医药及新药产业，启动建设生物医药产业园。积极发展新型纺织面料、新型建筑材料等新材料产业。拓展物联网技术应用领域，筹划设立福州经济技术开发区物联网园，扶持发展新大陆科技集团等一批物联网龙头企业，推动信息网络产业成为经济发展的新引擎。

　　四是创新引领，增强产业核心竞争力。引导创新要素向企业集聚，支持企业设立技术中心、研发中心、博士后工作站，加快发展行业技术创新中心、科技企业孵化器等公共创新服务平台，组织实施一批共性应用技术和瓶颈技术攻关，提高区域自主创新能力。大力推进工业化与信息化的融合，抓好中铝瑞闽等160项工业重点技改项目建设。依托“6·18”活动平台，推进项目、资金、技术、人才的对接。加强与在榕高校、科研院所的合作，开展科技人员服务企业行动，提升产学研合作水平。认真实施“人才强市”战略，大力培养和引进我市产业发展急需的高层次、高技能人才，发挥人才在产业发展中的带动和推动作用。

　　五是破解难题，切实为中小企业发展壮大提供良好服务。加快建设中小企业公共服务平台、信息服务网络和小企业创业基地，落实对小型微利企业的税收优惠政策。建立小企业金融服务体系，支持发展小额贷款担保公司，完善中小企业信用担保补偿和小企业贷款风险补偿机制。强化品牌带动，完善和落实甲供、甲控等政策措施，鼓励与支持企业拓展市场，推动名优地产品扩大市场占有率与影响力。

　　（三）把第三产业作为发展亮点加快培育

　　一是大力扶持现代服务业发展，培育新的经济增长点。坚持市场化、产业化、社会化的发展方向，促进服务业拓宽领域、增强功能、优化结构。大力发展面向民生的服务业，重点发展社区服务、家政服务、养老服务，满足人民群众多层次的需求。大力发展面向生产的服务业，重点发展金融、物流、信息、中介、代理、会计等服务，鼓励做大做强总部经济、楼宇经济。大力发展面向农村的服务业，重点发展产销、科技、信息和金融服务组织，加强农业生产的产前、产中、产后服务。

　　二是加快市场建设，发展新型商贸流通。建成海峡水产品、农副产品、汽车等三大交易中心，推进海峡钢贸城、海峡现代医药物流交易中心、奥特莱斯城市综合体、苏宁物流、海峡粮食加工物流园区等重大项目建设。加快发展品牌连锁、电子商务等现代营销业态，规划建设若干个新的城市综合体。设立福州保税港区。

　　三是着力做大会展业、旅游业，建设区域性会展中心城市和中国知名旅游目的地。建成海峡国际会展中心，办好中国文化用品商品交易会巡回展、香港时尚购物展等一批重大展会，争取每个月都有国家级或区域性展会在福州举办。大力推动高铁时代旅游业的跨越发展，全面提升旅游业，下大力气进一步打响“三坊七巷”、船政、昙石山、寿山石等闽都文化旅游品牌，加强对温泉休闲、生态山水、滨江滨海等福州特色旅游资源的宣传与推介，开发闽江游等一批上规模、上档次的旅游度假项目，打造中国重要的自然文化旅游中心城市和富有特色的旅游目的地。

　　四是加快发展服务外包和文化创意产业，打造对台服务外包示范城市和海西创意中心。拓展软件开发、系统应用和业务流程等领域服务外包，培育壮大动漫游戏等七大文化创意产业，推进海西动漫创意之都等项目建设，争取福州成为国家动漫产业基地。

　　五是增强消费拉动，在拓展城乡消费中壮大服务业。认真落实家电、汽车下乡及以旧换新等政策，繁荣城乡消费市场。拓展农村流通网络，推进“万村千乡”市场工程和“新网工程”建设。大力发展文化、信息、健身、培训等服务消费，鼓励发展网络购物、信贷消费等新型消费方式。整顿和规范市场经济秩序，加强价格监测调控、食品药品安全和信用体系建设，营造便利、安全、放心的消费环境。（四）把项目带动作为发展后劲提速增效

　　一是扩大投资规模，保持固定资产投资较快增长。在优化投资结构、提高投资效益的前提下，继续通过强投入培育形成一批新的经济增长点，进一步拉动经济增长、调优经济结构、增强发展后劲。初步安排2010年全社会固定资产投资2100亿元。在城镇固定资产投资中，基本建设投资达700亿元，占城镇投资的35%，增长52%；技改投资达680亿元，占城镇投资的34%，增长28%；房地产投资达500亿元，占城镇投资的25%，增长43%；其他投资达120亿元，占城镇投资的6%，增长20%。

　　二是全力推进重点项目建设，全面完成建设任务。初步安排市级重点项目323项，总投资5269.6亿元，年度计划投资619.5亿元。在铁路建设上，推进合福铁路福州段、向莆铁路福州段及可门、江阴、罗源湾北岸铁路支线等6项在建项目建设，动工建设火车北站新站房，力争完成投资35亿元。在高速公路建设上，推进福银高速公路福州南连接线等7项在建项目建设，动工建设京台线建瓯至闽侯高速公路福州段、绕城高速公路东南段，建成机场高速公路二期工程，力争完成投资60亿元。在能源建设上，动工建设福清核电3号机组，推进三山泽岐风电场、华能电厂三期等6项在建项目建设，力争完成投资90亿元。在轨道交通建设上，全线动工建设轨道交通1号线，力争完成投资35亿元。

　　三是扎实开展项目前期工作，持续生成一批带动力强的大项目。精心编制“十二五”发展规划和各重点专项规划，有计划、有重点地对经济社会发展带动力强的重大项目进行前瞻性的研究，深化项目规划、项目论证。加紧筹划和储备一批城市基础设施、产业发展、社会事业发展项目，突出策划一批符合国家产业政策和投资导向的生产性投资项目，成熟一个，实施一个，形成滚动推进的良好格局。做细做深项目前期工作，简化项目审批程序，力争大部分今年新开工项目在第二季度前动工建设。

　　四是增强财政金融保障作用，拓宽项目建设资金筹措渠道。依法依规加强税收征管，保持财政收入持续、均衡、稳定增长。提高财政科学化、精细化管理水平，加强部门预算管理、投资评审管理和政府采购等工作，扩大国库集中支付范围，集中财力保法定增长、保民生支出、保重点建设，合理控制政府性债务，提高财政资金使用效益。充分发挥市级投融资平台的带动作用，盘活存量资产，创新投资机制，拓宽融资渠道。加大政银企合作力度，支持企业发行债券或在境内外资本市场直接融资，鼓励发展风险投资，推行BOT、BT等建设项目融资方式。鼓励民间投资法律法规未禁止的行业和领域，发挥商会、行业协会作用，引导社会资金参与产业、基础设施和公益事业项目建设。

　　（五）把改革开放作为发展动力先行先试

　　一是突出综合实验，全面推进平潭开放开发。积极探索两岸共同合作具体模式，争取设立两岸合作海关特殊监管区，推进国际旅游商贸城等一批重大项目开发建设。加快建设适应大开发需求的基础设施，争取2010年第四季度建成平潭海峡大桥，2011年第三季度建成渔平高速公路，全面推进平潭环岛路、对台客货滚装码头建设，做好京台铁路平潭连接线、平潭二桥等项目前期工作，尽快启动22万伏进岛电力线路、江仔口22万伏变电站建设，加快完善供水、供气、排污、通讯等配套设施。科学合理开发利用海域、岸线等资源，打造生态旅游休闲岛。

　　二是突出市场导向，着力深化各项改革。稳步推进水价等资源性产品价格和污水处理、垃圾处理等环保收费改革。深化国有企业改革，全面整合盘活国有存量资产，培育市属百亿国企和上市储备资源。健全现代企业制度，完善企业法人治理结构，严格国有资产经营责任，促进国有资产保值增值。增强民营经济发展活力和竞争力，在招投标、政府采购等方面提供同类政策待遇，完善融资、土地使用、公共技术服务等方面的扶持政策与服务。拓展公共资源市场化配置工作，规范与完善政府采购、工程、土地、产权等领域的电子化交易。继续推进污水处理、垃圾处理、园林绿化、道路保洁等市场化运作、产业化经营。认真实施政府机构改革、乡镇机构改革和事业单位分类改革，进一步加快转变政府职能、规范机构设置、理顺职责关系、优化组织结构。

　　三是突出保份额、调结构、促平衡，积极拓展对外经贸。加大对重点外贸企业扶持力度，扩大出口信用保险覆盖面。提高自主品牌和高技术含量、高附加值产品出口比重，鼓励加工贸易转型升级。推进科技兴贸创新基地建设，着力培育光电、纺织、显示器及配件等省级重点产业出口基地。支持企业“走出去”拓展海外市场，有效应对国际贸易和技术壁垒。加快电子口岸建设，进一步提高口岸通关效率。鼓励企业引进先进技术、关键设备，扩大重要资源、原材料进口。强化重大外资项目协调推进机制，加强对龙头项目、产业链上下游配套项目和填补产业空白项目的招商，促进重大招商项目尽快落实落地。大力争取央属企业将产业龙头项目布局在我市，认真抓好中储粮、神华等一批大项目建设。不断提高“5·18”活动办会水平与实效，办好第六届亚太经合组织技展会等活动，积极参加第十四届“9·8”投洽会等国内外经贸活动。进一步支持现有企业增资扩产，鼓励在榕上市企业扩大投资。大力实施“回归工程”，充分发挥行业协会、异地商会的桥梁纽带作用，更加积极地引导海内外乡亲回乡投资兴业。

　　四是突出打响海峡品牌，不断拓展榕台合作和区域合作。在电子信息、汽车制造、软件、动漫游戏、光机电一体化、精密机械等领域，积极承接台湾先进制造业转移，筹划建设东南IC制造业基地。推进榕台农业合作，办好台湾农民创业园等海峡两岸（福州）农业合作试验区八大园区。发挥区位优势，提升榕台贸易水平，争取台湾水产品市场准入在福州先行先试。抓住海峡两岸签署金融合作监管备忘录的机遇，吸引台湾金融机构来榕投资设点。争取开展外地临时来榕人员赴台旅游就地办证试点，打造榕台互动旅游黄金通道。积极拓展榕台“三通”，争取实现空中货运直航，争取组建两岸邮轮公司，争取开通海峡快捷走廊，争取建成两岸邮政枢纽中心。建立榕台交流常态化机制，拓展榕台科技、教育、文化、卫生、体育等领域全方位、多层次的交流，支持办好旅台福州同乡社团恳亲大会等活动，推动与台北、基隆等城市的对口交流。全面深化与香港、澳门在对外经贸、人才交流等方面的合作。进一步落实侨务政策、涵养侨务资源、发挥侨智侨力，巩固和拓展对外合作渠道。提升闽东北五市和闽浙赣皖福州经济协作区合作水平，加快构建区域共同市场。加强国内外友城合作，配合办好第六届泛珠三角区域合作发展论坛暨经贸洽谈会。继续实施四川地震灾区对口援建项目，认真做好对口支援等工作。　（六）把城乡建设作为发展环境优化提升

　　一是强化规划控制，优化城市空间布局。积极推进城市总体规划和土地利用总体规划报批工作，加强总体规划与综合交通、历史文化名城保护、城市抗震等专项规划的相互衔接。强化规划的指导性、约束性、权威性，扩大规划覆盖面，深化长乐、闽侯、连江、平潭等重点开发区域和东部新城、晋安新城控制性详规，实现中心城区近期发展及重点建设区域的控制性详规全覆盖。完成闽江北港等重点区域城市设计。以规划为龙头，积极稳妥推进行政区划调整，加快中心城市东扩南进、沿江向海发展步伐，构建福州大都市区。

　　二是提升城区建设水平，改善城市整体形象。坚持高起点、高标准、高层次、高水平，加快推进东部新城、海峡金融商务区、闽江北岸中央商务区等重点区域建设，成片开发火车南北站周边片区和晋安新城鹤林片区，提升五四北新店片区，完善金山新区服务配套。推进旧城区及危旧房（棚屋区）成片改造，加快实施首批500万平方米改造任务。加快琅岐、长乐等沿江地区开发步伐，动工建设琅岐闽江大桥、琅岐环岛路，争取启动建设城区连接琅岐的快速通道。

　　三是打造宜居环境，凸显城市特色魅力。改造提升闽江北港江滨沿线景观，美化江滨环境。强化市区内河截污疏浚，实施白马河、晋安河（光明港）等主要内河综合整治，加快南台岛12条内河整治，结合旧城改造推进磨洋河整治。建设新儿童公园，实施闽江北港绿化建设，加强道路绿化、小区绿化、立体绿化，持续巩固创卫、创园、创模、创绿成果，提高城市绿化、彩化、美化、香化水平。

　　四是加强基础设施建设，增强城市承载功能。新改扩建南站南路等一批市区主次干道和断头路、丁字路，建成鼓山大桥，加快林浦大桥、螺洲大桥、淮安大桥等桥梁建设。扩大公交线路覆盖范围，落实公交路权优先，新增500辆公交车。加快建设一批城区停车泊位。建成琴亭湖，推进闽江南港防洪工程建设，建设魁岐排涝站二站。全面实施城区主干电网及电缆下地、液化天然气利用、自来水一户一表改造等工程。

　　五是持之以恒开展整治工作，提高城市管理水平。健全城市管理问责机制、长效机制，加强依法管理、文明管理，深入开展城市管理专项整治行动。综合整治提升闽江北港，持续开展非法采砂、“连家船”、废弃码头等水域清理整治，实施货运船舶禁航。开展城区交通整治。加强鼓岭综合治理，精心打造城市“后花园”。坚持依法依规、有理有节、敢于碰硬、严格执法，坚定不移集中清理城区违法建设，进一步扬福州正气、展福州新貌。

　　六是积极推进城镇化，加快城乡一体化发展。围绕提升中心城区、拓展南北“两翼”、推进东西“两片”的区域发展格局，加快构建闽江口城镇群。支持闽侯、连江、闽清、罗源、永泰等县城改造建设，择优发展青口等10个示范性小城镇。推动青岛啤酒、东南电化、耀隆化工等城区工业企业按照产业布局导向，向周边县（市）搬迁，支持各县（市）发展特色产业，做大做强县域经济。稳步推进进城农民融入城镇，逐步实现农民工在劳动报酬、子女就学、公共卫生、住房租购以及社会保障等方面与城镇居民享有同等待遇。放宽户籍落户条件，推动在城镇稳定就业和居住的农民有序转变为城镇居民。

　　（七）把生态文明作为发展取向常抓不懈

　　一是大力推进节能减排攻坚，促进经济社会可持续发展。认真实施“十城万盏”半导体照明应用等重点节能工程，推进钢铁企业高炉余压发电、电力企业及纺织企业变频节能技术改造等80家企业重点节能项目建设。加强城区禁煤、电力企业脱硫监管，支持和推动闽清陶瓷企业改燃气降耗减排等技术改造，切实削减大气污染物排放量。加快推进污水、垃圾处理设施建设，基本建成连坂、浮村污水处理厂，建设第二垃圾焚烧发电厂，确保每个县（市）至少建成一座污水处理厂和一座垃圾处理厂。

　　二是强化环境保护和生态建设，促进人与自然和谐发展。严格执行建设项目节能评估审查和环境影响评价、环保“三同时”等制度，强化合同能源管理、重点污染源在线监控。加强水、气、声、渣等各类环境问题整治，突出抓好闽江南北港、敖江、龙江、大樟溪等重点流域水环境综合整治，有效控制石板材、畜禽养殖等污染，基本建成沿江生活污水、垃圾处理设施，实现工业污染全面稳定达标排放，确保生活饮用水水质安全。抓紧编制海洋环境保护规划，实施“碧海银滩”工程，开展罗源湾养殖清理，强化海洋环境综合整治。继续治理青山挂白，加强沿海防护林、生态公益林保护与建设，推进环福州绿色屏障建设。强化闽江口湿地等自然保护区、风景名胜区、生态功能区保护，加大生态县（市）、环境优美乡镇、生态示范村创建力度。完善生态环境补偿机制，提高森林生态效益补偿标准。

　　三是提高资源节约集约利用水平，促进资源节约型、环境友好型社会建设。修编完善区域用海规划、岸线保护利用规划，优化配置、集约利用土地和海域、岸线等资源。充分运用市场机制，通过二次开发、收购储备等措施积极盘活存量土地，鼓励开发利用地下空间，提高单位土地投资强度与产出效益，坚决依法处置闲置土地、岸线。加强河砂、温泉等资源有效保护与合理开发。建立完善再生资源回收体系，促进城市生活垃圾资源化利用和重点行业废弃物再利用。加快发展循环经济，支持企业开展清洁生产和资源综合利用，创建青口循环经济示范区和14家省级循环经济示范企业。加快建设以低碳排放为特征的产业体系和消费模式，发展低碳经济，倡导低碳生活。（八）把社会事业作为发展保障协调推进

　　一是优先发展教育事业，为经济社会发展提供智力支撑和人才保障。提高办学水平，全面完成义务教育“双高普九”任务，加快普及高中阶段教育。深入实施素质教育，促进义务教育均衡发展，推动城市优质教育资源向农村延伸。新建三江口高级中学等7所中小学，改扩建10所中小学，建设100所以上农村义务教育标准化学校。加快发展职业教育，推进公共实训基地建设，实现所有县（市）都有县级职教中心。积极盘活高等院校市区资源，进一步提升高等教育办学质量。加强对学前教育的管理，规范发展民办教育、特殊教育、成人教育、老年教育等各级各类教育。加强教师队伍建设，提高师德师风水平，打造名师群体。

　　二是做好卫生、计生、体育等工作，切实提高全民健康水平。推进医药卫生体制改革，加大公共卫生事业发展力度，完善市、县（市）区、乡镇（街道）三级突发公共卫生事件应急机制、疾病预防控制、医疗救治和卫生监督体系。加快实施国家基本药物制度，开展医疗机构药品集中招标采购。加快公立医院改革试点，鼓励社会力量兴办医疗机构，在金山新区、东部新城启动建设2所三级综合性医院。全面提升乡镇卫生院医疗水平，推进社区卫生服务机构标准化建设。深入开展群众性爱国卫生运动，继续做好甲型H1N1流感等重大疾病防控工作。加强医患纠纷预防、调处、化解工作，积极构建和谐医患关系。坚持计划生育基本国策不动摇，统筹解决人口问题，加强人口计生教育宣传，强化社会抚养费征收，逐步解决出生人口性别比失衡等问题，认真做好第六次全国人口普查工作，进一步稳定低生育水平、提高出生人口素质。配合办好第五届全国特奥会，争取在第十四届省运会上创出佳绩，积极申办第八届全国城运会，推动全民健身活动深入开展。

　　三是繁荣发展文化事业，丰富人民群众文化生活。动工建设市图书馆等一批体现省会城市水平的文化设施，配合抓好省科技馆建设，争取建成市工人文化宫等项目。加大农村文化基础设施建设力度，进一步健全完善农村、社区等基层基础公共文化服务体系，提高群众喜闻乐见的文化产品供给能力。加快文化体制改革步伐，深化公益性文化事业单位运行机制改革，推进经营性文化单位转企改制。加强文艺精品创作，开展首届茉莉花文艺奖评选，打造海峡两岸合唱节等特色文化品牌。强化历史文化名城和文化遗产保护，扎实推进“三坊七巷”等一批重大保护修复工程和涉台文物保护利用项目建设，彰显闽都文化深厚底蕴，提升城市文化品位。加强公民思想道德建设，广泛开展城乡志愿服务活动，扎实推进新一轮创建全国文明城市工作。

　　（九）把改善民生作为发展根本落实到位

　　一是拓宽就业渠道，促进充分就业。实施积极的就业政策，争取城镇新增就业14.5万人，转移农业富余劳动力4万人。以创业带动就业，完善以创业培训、项目开发、融资服务、创业孵化等为主要内容的创业服务体系。多渠道开发公益性就业岗位，努力确保就业困难家庭“一户一就业”。加强校企合作，强化农村劳动力就业培训、技能培训，拓展劳务协作网络，搭建职业教育与企业用工需求对接服务平台，积极解决企业结构性、阶段性缺工问题。实施大学生服务基层就业项目，推进大学生毕业见习基地建设，引导和鼓励高校毕业生面向企业、社区、农村就业。完善劳动关系三方协调机制，积极开展创建劳动关系和谐企业与工业园区活动。

　　二是规范收入分配秩序，提高城乡居民收入水平。深化分配制度改革，着力提高居民收入在国民收入分配中的比重、劳动报酬在初次分配中的比重。认真落实提高农民收入、企业退休人员基本养老金和部分优抚对象待遇的各项政策。落实义务教育学校、公共卫生与基层医疗卫生事业单位绩效工资改革。推动工资集体协商和工资支付保障制度建设，促进企业落实最低工资制度与工资正常增长机制。

　　三是完善社会保障体系，筑牢民生安全网。扩大“五险”覆盖面，推进跨统筹地区养老保险、医疗保险关系无障碍转移接续，稳步实施城镇职工基本医疗保险和工伤保险市级统筹。巩固和发展新型农村合作医疗和城镇居民基本医疗保险，实施新型农村社会养老保险试点。启动市民卡工程建设，先期保证社保应用，两年内实现一期工程150万张发卡量。完善最低生活保障、临时救助、生活补助和社会捐助相结合的城乡社会救助体系，发展社会福利、救助和慈善事业，为年满70周岁、生活不能自理且在市区无子女照顾的低保老人、重点优抚对象、“五保”老人、革命“五老”人员提供无偿居家养老服务，逐步扩大城乡医疗救助范围。完善和落实征地拆迁补偿政策，切实维护被征地、被拆迁群众的合法权益。加强救灾救济和老龄、妇女儿童、残疾人等工作，建设应急救灾物资储备库等一批民政设施。

　　四是稳定商品房价格，促进房地产市场健康发展。认真实施保障性安居工程，开展住房公积金贷款支持保障性住房建设试点，加快廉租房、经济租赁房、限价房建设步伐，扩大廉租房保障范围。增加中小户型、中低价位普通商品住房用地供给，支持居民自住和改善型住房消费，抑制投机性购房，遏制房价过快上涨。规范发展住房二级市场，盘活住房租赁市场。整顿规范房地产市场秩序，加大对囤地不建、捂盘惜售、囤积房源、哄抬房价等违法行为的查处力度。

　　五是办好惠民实事，让改革发展成果惠及最广大人民群众。实施中小学校舍安全工程，改造10万平方米、重建15万平方米中小学校舍；改善福州盲校、聋哑学校等特殊教育学校办学条件。免费为城乡居民提供预防接种等九类基本公共卫生服务；选派150名医师帮扶基层医疗卫生机构；培训3900名乡村医生；建设20所示范性村卫生室。改造完善30个乡镇综合文化站；争取一村一月放映一场电影；新建、改建农家书屋500家；新建400个农民体育健身工程点、8个农民体育健身活动中心。启动建设海峡妇女儿童活动中心。新建、改扩建一批农村敬老院，新建30个社区居家养老服务点；为800名贫困的白内障患者免费实施复明手术；新建10所公办“福乐家园”，资助300名残疾人居家托养；对200名贫困学龄前残疾儿童给予每人1万元康复训练补助。继续治理“餐桌污染”，建设“食品放心工程”，净化市场环境。（十）把安定稳定作为发展大局齐抓共管

　　一是深化平安建设，履行好“保一方平安”的职责。健全维护稳定、打击违法犯罪的长效机制，有重点、有成效地组织开展专项打击整治活动，解决市民反映突出的入室盗窃、盗窃电动自行车等治安难点问题，巩固打“两抢”、场所涉毒等专项整治成果，努力实现人民群众对社会治安的满意率稳中有升。加强公安信息化建设，完善城市治安现代防控体系，强化网络监管。推进公安执法规范化，构建和谐警民关系。严格落实安全生产责任，深化安全生产“三项行动”、“三项建设”，在消防、道路交通、危险化学品等重点行业和领域继续开展安全生产专项整治行动，坚决整改重大安全生产事故隐患，有效遏制重特大事故发生。

　　二是坚持以人为本，提升社会管理水平。加强社区建设与管理，增强综合服务功能，积极创建和谐社区。加大流动人口管理力度，完善“一站式”服务。实施关爱工程，强化对特殊未成年人群体的教育管理和服务救助。推进社区矫正试点和青少年事务社工试点工作。依法管理各类非政府组织。扩大基层民主和群众自治。认真贯彻落实党的民族宗教政策，依法管理宗教事务，扶持少数民族地区加快发展。积极开展双拥共建活动，加强国防动员、民兵预备役、海防、人防等工作，帮助驻榕部队建设一批“四个一好”生产生活项目。

　　三是妥善化解社会矛盾纠纷，确保社会和谐稳定。通过集中接访、领导下访、专门约访等形式，及时化解影响社会和谐稳定的矛盾和问题。认真办理便民呼叫中心12345系统受理的诉求件，深入开展“三排查一促进”等活动。加强普法教育、法律援助、法律服务、司法鉴定等工作，引导群众依法有序表达利益诉求。发挥人民调解作用，及时调处民间纠纷。规范突发事件应急处置办法，依法妥善处置群体性事件。

三、强化责任意识，建设服务型政府

　　海西建设千帆竞发、百舸争流。面对难得的历史机遇，我们要在已有的发展基础上，以更快的发展速度力争走在全省前列，加快建设环境优美、居住舒适、人才集聚、辐射带动力强的省会中心城市，在海西建设全局中更好地树立省会窗口形象、发挥省会带动和辐射作用。市政府在推进发展中将坚持服务为本、责任为先，切实做到：

　　忠诚履责。围绕发展讲政治、讲大局、讲党性，不折不扣地落实好中央、省和市委的各项决策部署。在其位、谋其政、尽其责，始终牢记发展第一要务，坚定不移地盯紧发展、谋划发展、推动发展，以经济社会发展的实际成果体现对党忠诚、对人民负责。依法行政、科学行政、阳光行政，加强行政立法，强化执法监督，规范执法行为，完善政府科学民主决策机制，推进政务公开、政府信息公开。认真执行人大决议决定，依法接受人大及其常委会的法律监督、工作监督，主动接受政协的民主监督，自觉接受群众监督、舆论监督，确保人民赋予的权力始终用来为人民谋利益。

　　尽心尽责。政府工作重在落实。必须在真抓实干中尽责，少说多干、低调务实、不搞虚功，力求做一件事象一件事、办一件事成一件事。在勤政为民中尽责，勤勤恳恳，兢兢业业，始终深怀爱民之心、恪守为民之责、谋划富民之策，从人民群众的衣食住行、安危冷暖入手，想民之所想，急民之所急，集中财力多办惠民利民的实事好事。在提速增效中尽责，加快政府运作节奏，切实精减会议、文件，制定实施转变政府职能、提高行政审批效率的一揽子意见，推进以办公自动化、网上审批及效能监察等为重点的电子政务建设。在优化服务中尽责，深化机关效能建设，积极开展“十佳市级办事窗口”评选等活动，坚决奖优罚劣、惩庸治懒，促进各级政府和政府各部门满腔热情地支持企业发展，尽心尽力地帮助企业、群众排忧解难。

　　勇于负责。始终保持创新创业的朝气，更加注重用改革的办法、创新的理念解决发展中的问题，只要对发展有利、对事业有利、对人民群众有利的事，就大胆试、主动闯，努力突破阻碍发展的各种障碍。始终保持攻坚克难的勇气，勇于战胜困难，敢于担当风险，该碰硬的坚决攻坚，该拍板的大胆决断，遇到矛盾不回避、面对责任不推诿，不断在破解难题中提高应对复杂局面、驾驭市场经济的能力与水平。始终保持雷厉风行的锐气，对工作坚决一抓到底，确保落实到位，确保政令畅通，增强政府的执行力、公信力。

　　敢于问责。把重大决策部署的执行情况，重点工作、重大项目的落实情况，群众关注热点难点问题的解决情况作为问责重点，严格绩效管理，强化行政问责，切实做到有岗就有责，责随职走、心随责走。坚持从严治政，对干部进一步严格要求、严格教育、严格管理、严格监督，规范行政权力运行，坚决治理行政不作为、乱作为的行为。严格执行廉洁从政各项规定，强化行政监察、审计监督，加强公务接待、公车使用和公款出国（境）等管理，切实纠正损害群众利益的不正之风，严肃查处违纪违法案件，完善权力阳光运行系统，推进惩治和预防腐败体系建设，时刻保持清正廉洁、艰苦奋斗的本色。

　　各位代表，站在海西建设新的起点上，展现在我们面前的是福州更加美好的未来。机遇不能失去，困难要敢于面对。在新的一年，我们要紧密团结在以胡锦涛同志为总书记的党中央周围，在省委、省政府和市委的正确领导下，高举中国特色社会主义伟大旗帜，以邓小平理论和“三个代表”重要思想为指导，深入贯彻落实科学发展观，抢抓机遇、开拓奋进，满怀激情、加快发展，为开创福州又好又快发展的新局面，为发挥福州在海西建设全局中的带动和辐射作用而努力奋斗！
